# Supplementary material for: PBRM1 acts as a p53 lysine-acetylation reader to suppress renal tumor growth
Source: Nat Commun. 2019 Dec 20;10:5800. doi: 10.1038/s41467-019-13608-1 (PMC6925188; doi:10.1038/s41467-019-13608-1)
Supplement: Supplementary file 1 — Supplementary Information [file 41467_2019_13608_MOESM1_ESM.pdf]

PBRM1 acts as a p53 lysine-acetylation reader to suppress renal tumor growth

Cai et al.

Supplementary figures:

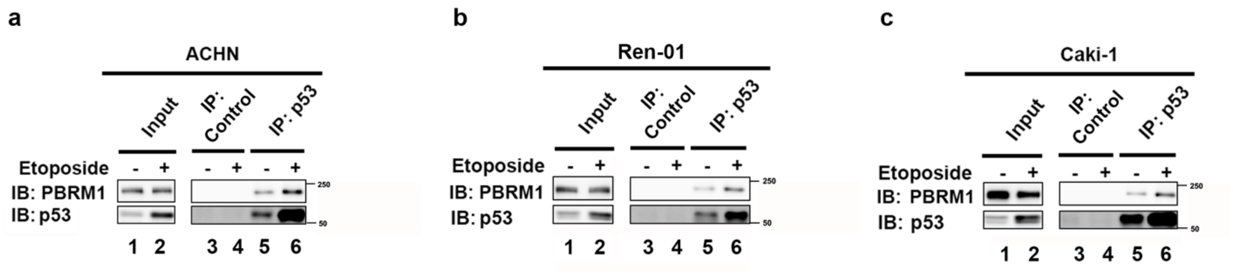

Supplementary Figure 1. Endogenous PBRM1 and p53 physically associate in kidney cancer cells.

ACHN (**a**), Ren-01 (**b**) and Caki-1 (**c**) cells were treated with vehicle (DMSO) or etoposide (50 μM) for 8 h and harvested for immunoprecipitation with control IgG and p53 antibodies. The bound PBRM1 and p53 were examined by immunoblots. Source data are provided as a Source Data file.

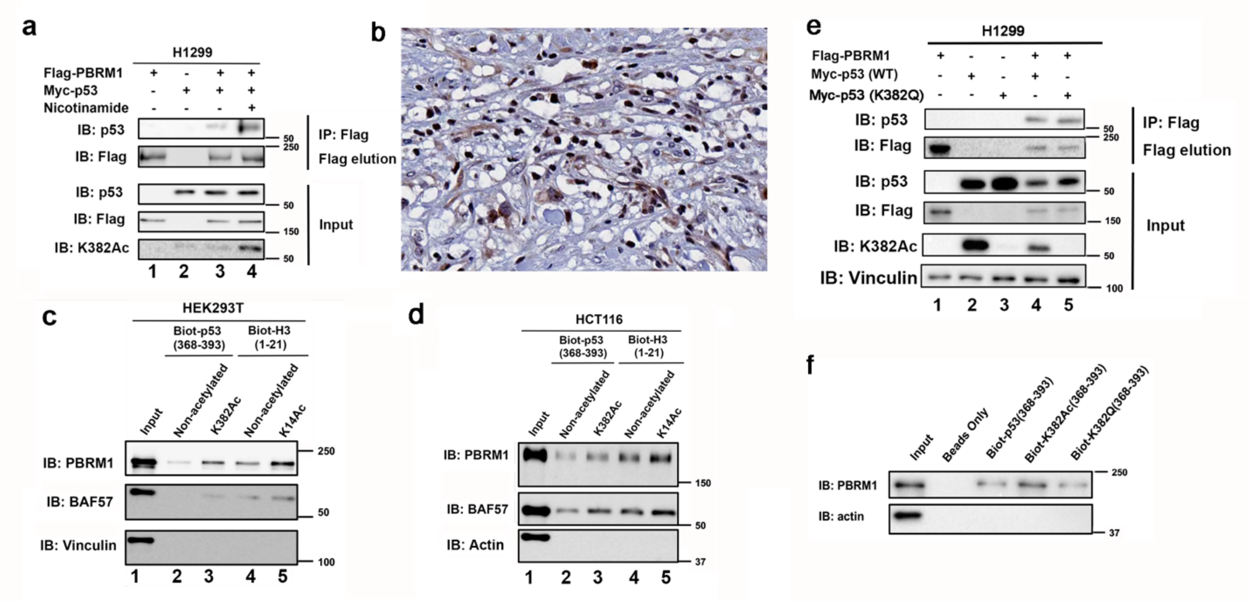

Supplementary Figure 2. Acetylation on K382 enhances p53's interaction with PBRM1 and it naturally occurs in renal tumors.

a. Vectors, Flag-PBRM1 or Myc-p53 were transfected into H1299 cells as indicated, and treated with 5 μM nicotinamide or vehicle. Lysates were used for anti-Flag immunoprecipitation and 3X Flag peptide elution. The inputs and eluates were immunoblotted with the indicated antibodies. **b.** A representative picture of immunohistochemical analysis of human ccRCC tumors with anti-K382Ac antibody. **c**, **d**. Biotinylated p53 peptides with lysine acetylation at the indicated sites were incubated with lysates from HEK293T cells (**c**) or HCT116 cells (**d**). The peptides were pulled down with streptavidin beads and the associated proteins were analyzed by immunoblots. e. Flag-PBRM1, wildtype or K382Q mutated Myc-p53 were transfected into H1299 cells with indicated combinations. Lysates were used for anti-Flagimmunoprecipitation followed by Flag peptide elution. The inputs and eluates were immunoblotted with the indicated antibodies. f. H1299 cell lysates were incubated with indicated biotinylated p53 peptides. The peptides were pulled down with streptavidin beads and the associated protein was immunoblotted with indicated antibodies. Source data are provided as a Source Data file.

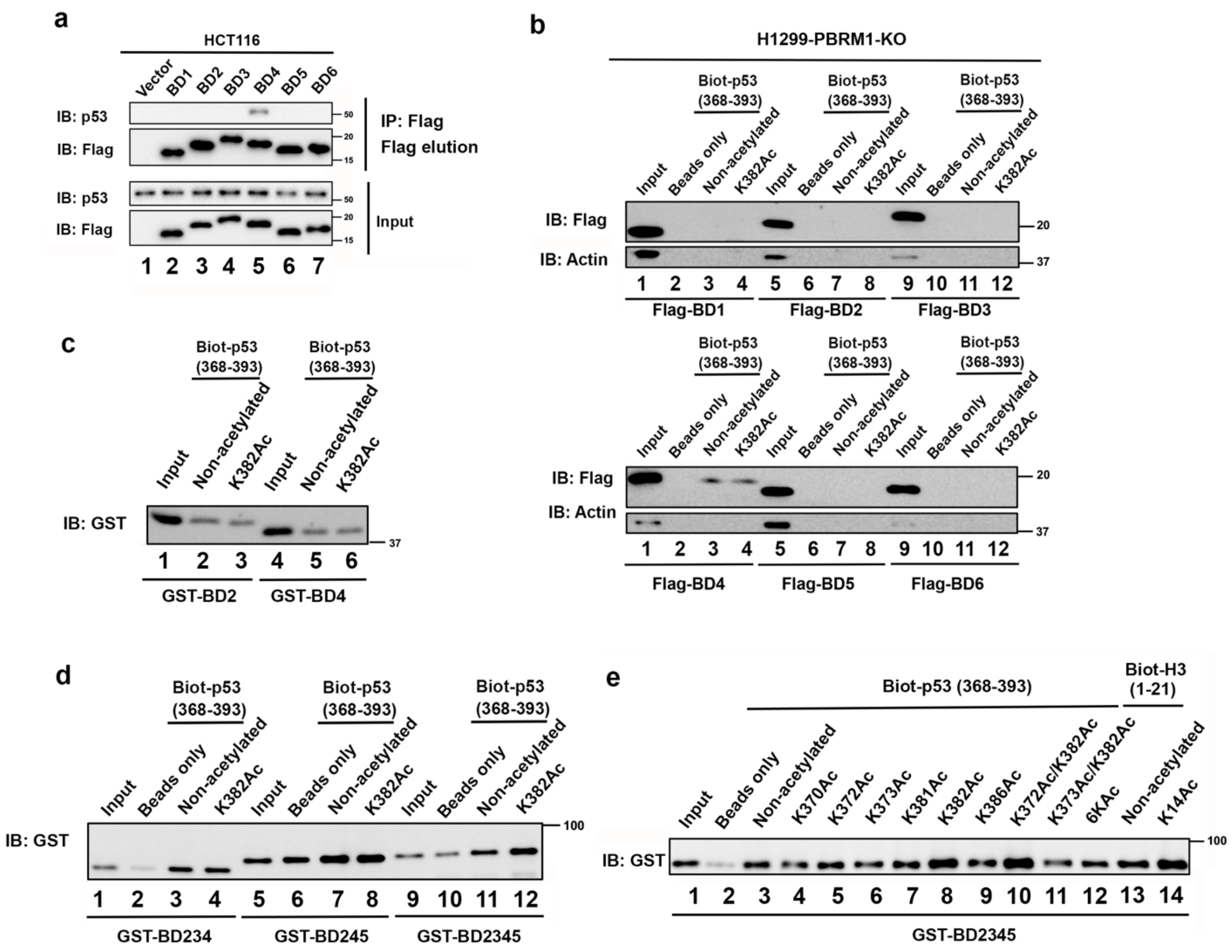

Supplementary Figure 3. Bromodomain 4 of PBRM1 is critical for binding p53.

a. HCT116 cells were transfected with vector or individual Flag-PBRM1 bromodomains and treated with 50 μM etoposide for 8 h. Lysates were subjected to immunoprecipitation with Flag-M2 beads. The inputs and eluates were analyzed by immunoblots. **b**. H1299 PBRM1 KO cells were transfected with the individual Flag-PBRM1 bromodomains as indicated. Lysates were incubated with biotinylated p53 peptides with lysine acetylation at the indicated sites. The peptides were pulled down with streptavidin beads and the associated proteins were immunoblotted with the indicated antibodies. **c**, **d**, **e**. Purified GST-PBRM1 BD2 and BD4 (**c**), bromodomain combinations (**d**) or BD2345 (**e**) were incubated with biotinylated p53 peptides with lysine acetylation at the indicated sites. The peptides were pulled down with streptavidin

beads and the associated proteins were immunoblotted with anti-GST antibody. Source data are provided as a Source Data file.

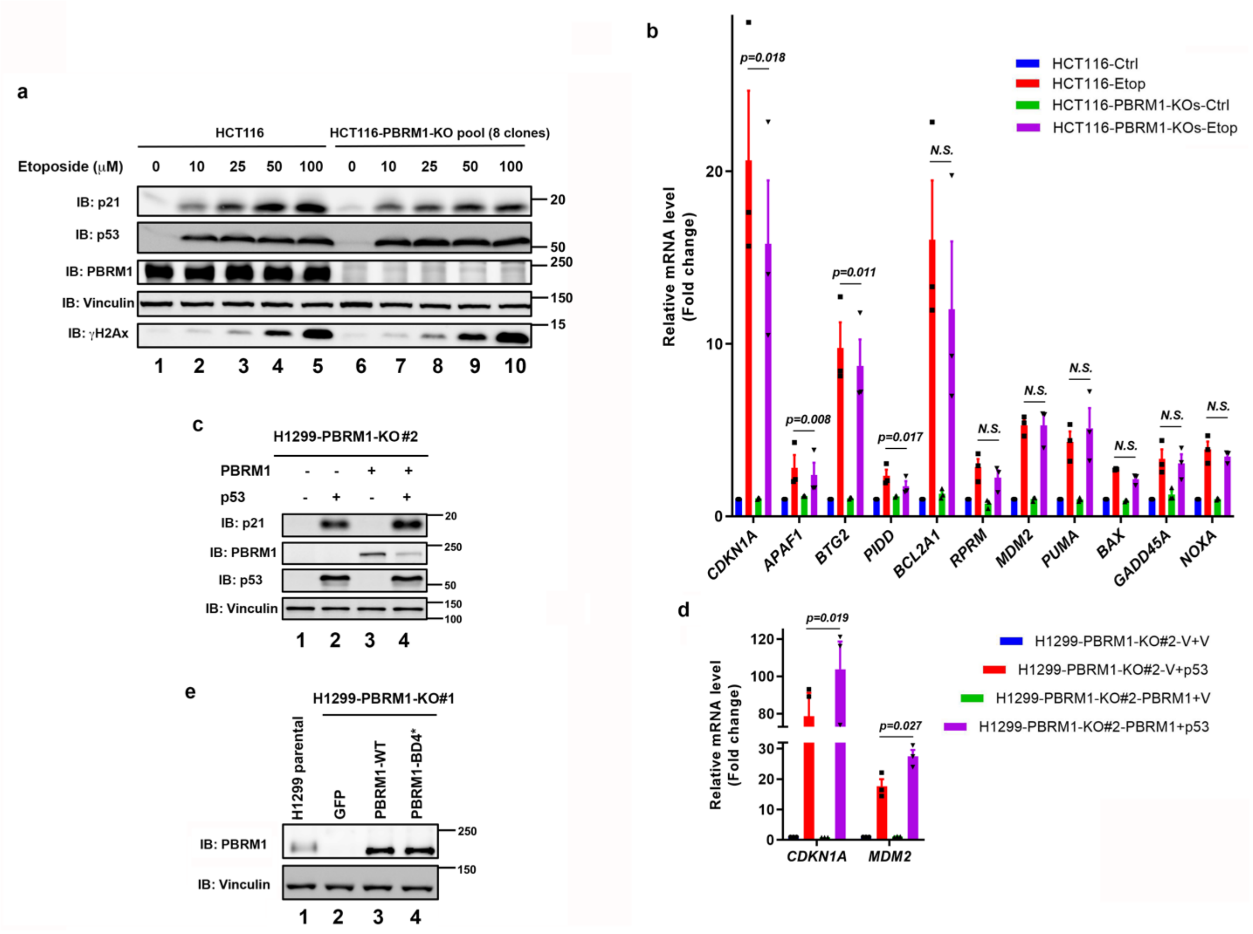

Supplementary Figure 4. PBRM1 is required for full p53 transcriptional activity.

a, **b**. HCT116 parental and PBRM1 KO cells (combination of eight clones) were treated with vehicle or etoposide at the indicated doses for 24 h, and lysates were analyzed by immunoblots (**a**). RNA was extracted and reverse transcribed from the indicated cells treated with 50 μM etoposide. Gene expression was measured by qPCR (**b**), and data are shown as mean ± SEM. p-values were calculated using the paired two-tailed Student's t-test. **c**, **d**. Vectors, Flag-PBRM1 and Myc-p53 were transfected into H1299 PBRM1-KO#2. Cells were harvested for immunoblot analysis (**c**) and RT-qPCR (**d**). N= 3 biologically independent samples. **e**. GFP, wild-type or BD4* mutant PBRM1 were stably expressed in H1299 PBRM1-KO#1. Lysates were analyzed via immunoblot to examine PBRM1 expression. Source data are provided as a Source Data file.

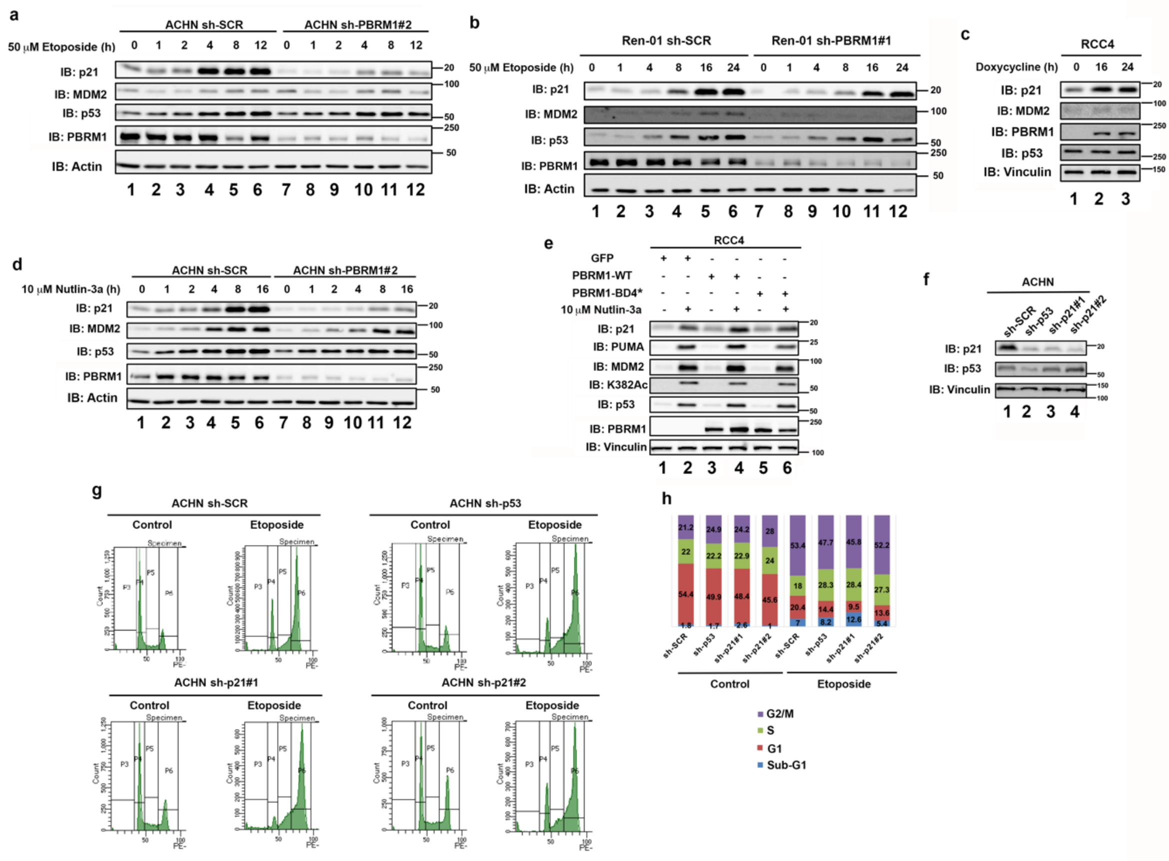

i

% of total cells (mean ± SEM)	Sub-G1	G0/G1	S	G2/M
ACHN sh-SCR-Ctrl	1.8±0.5	54.4±3.7	22.0±3.8	21.2±1.1
ACHN sh-PBRM1#1-Ctrl	0.9±0.2	50.2±4.0	27.5±5.9	20.7±9.5
ACHN sh-PBRM1#2-Ctrl	1.4±0.2	49.3±0.7	21.6±3.2	26.4±4.1
ACHN sh-p53-Ctrl	1.7±0.2	49.9±3.3	22.2±3.1	24.9±1.8
ACHN sh-p21#1-Ctrl	2.6±1.2	48.4±1.4	22.9±2.9	24.2±2.6
ACHN sh-p21#2-Ctrl	1.0±0.1	45.6±2.6	24.0±2.7	28.0±5.2
ACHN sh-SCR-Etop	7.0±2.5	20.4±0.7	18.0±1.6	53.4±5.3
ACHN sh-PBRM1#1-Etop	3.4±0.5	15.2±3.3	27.1±7.1	53.6±10.6
ACHN sh-PBRM1#2-Etop	6.2±0.9	12.5±2.0	22.0±5.8	57.9±8.8
ACHN sh-p53-Etop	8.2±2.1	14.4±1.8	28.3±6.3	47.7±9.6
ACHN sh-p21#1-Etop	12.6±4.1	9.5±1.6	28.4±6.0	45.8±11.6
ACHN sh-p21#2-Etop	5.4±0.8	13.6±0.9	27.3±6.3	52.2±7.0

Supplementary Figure 5. PBRM1 is critical for the p53 signaling pathway and its biological function in ccRCC cells.

a. ACHN control and PBRM1 shRNA #2 knockdown cells were treated with 50 μM etoposide for the indicated times. Lysates were analyzed via immunoblots. **b**. Ren-01 control and PBRM1 shRNA #1 knockdown cells were treated with 50 μM etoposide for the indicated times. Lysates were analyzed by immunoblots. **c**. RCC4 cells containing inducible PBRM1 were treated with doxycycline for the indicated times, and lysates were immunoblotted. **d**. ACHN control and PBRM1 shRNA #2 knockdown cells were treated with 10 μM Nutlin-3a for the indicated times. Lysates were analyzed via immunoblots. **e**. RCC4 cells stably expressing GFP, wild-type or BD4* mutant PBRM1 were treated with 10 μM Nutlin-3a. Lysates were analyzed via immunoblots. **f**, **g**, **h**. ACHN control, p53 and p21 shRNA knockdown cells were harvested for immunoblots (**f**) or treated with vehicle or 50 μM etoposide for cell cycle analysis. Representative data is shown in **g**. Quantification of cell cycle phase is shown in **h** and **i** as mean ± SEM. N= 3 biologically independent samples. Source data are provided as a Source Data file.

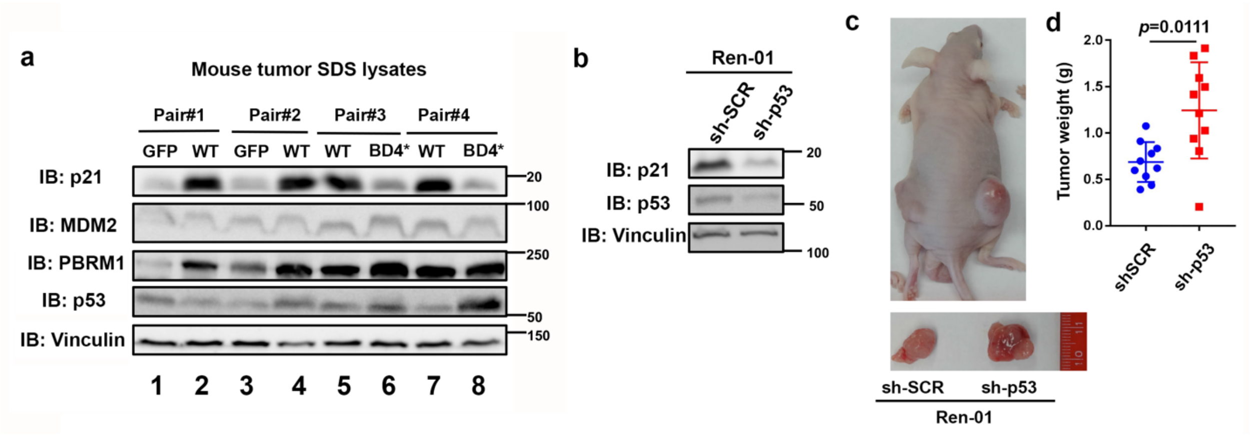

Supplementary Figure 6. Suppression of p53 promotes tumor growth in Ren-01 cells.

a. Protein was harvested from tumors formed by Ren-01 PBRM1 KO cells expressing GFP, wild type PBRM1 (WT) or BD4* mutant PBRM1 (AA) in nude mice. Lysates were analyzed by immunoblots. **b**, **c**, **d**. Ren-01 control and p53 shRNA knockdown cells were harvested for immunoblots (**b**) and used in nude mice xenograft experiments. Tumors were excised and weighed, and data are shown as mean ± SEM (**d**). p-values were calculated using the paired two-tailed Student's t-test. N= 10 biologically independent mice. Representative photographs of nude mouse (**c**, top) and tumors (**c**, bottom). Source data are provided as a Source Data file.

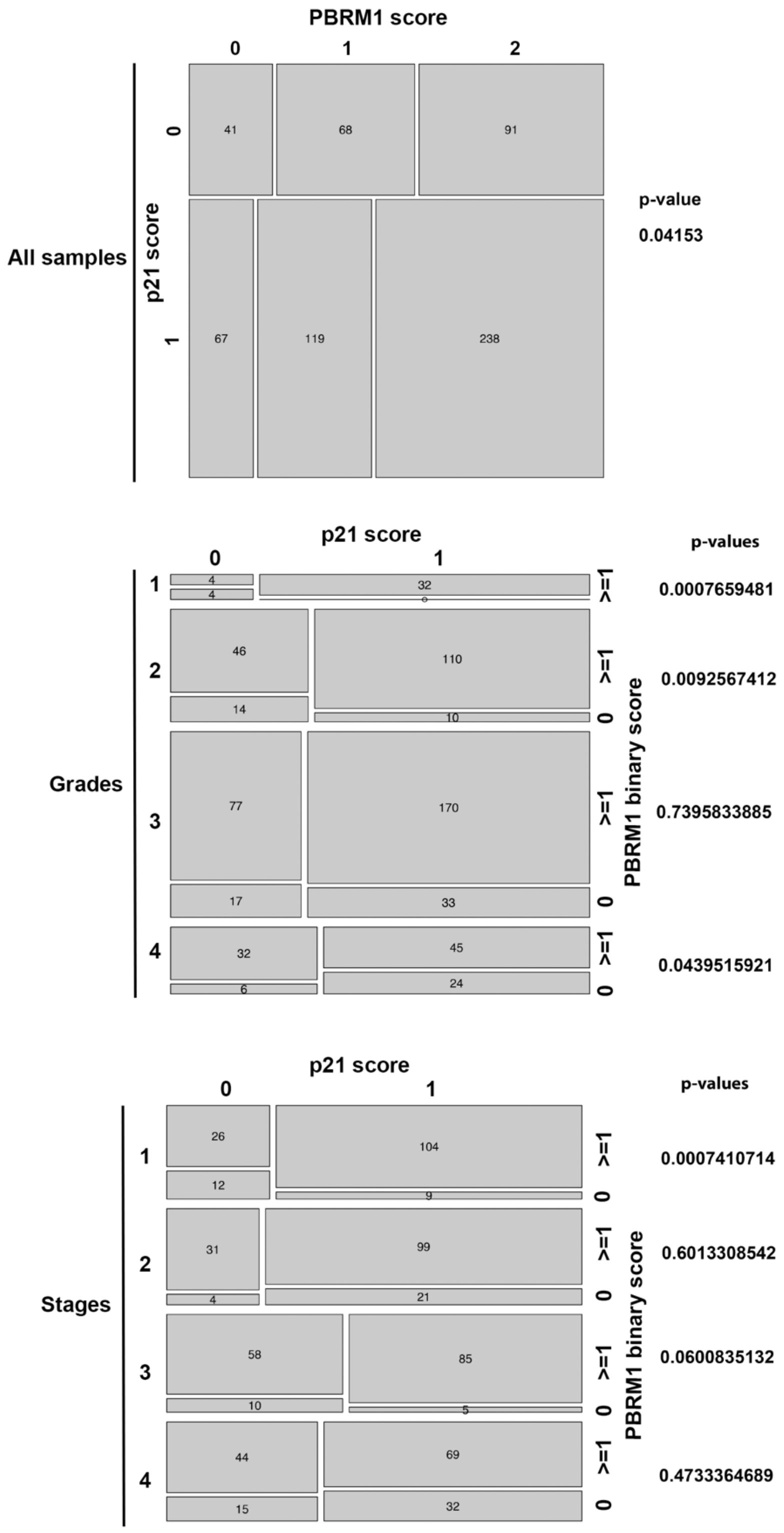

PBRM1 score
0
1
2
p21 score
0
1
41
68
91
67
119
238
All samples
p-value
0.04153
p21 score
0
1
p-values
Grades
1
2
3
4
4
4
32
0
46
110
14
10
77
170
17
33
32
45
6
24
>=1
0
PBRM1 binary score
0.0007659481
0.0092567412
0.7395833885
0.0439515921
p21 score
0
1
p-values
Stages
1
2
3
4
26
104
12
9
31
99
4
21
58
85
10
5
44
69
15
32
>=1
0
PBRM1 binary score
0.0007410714
0.6013308542
0.0600835132
0.4733364689

Supplementary Figure 7. PBRM1 loss and p21 loss correlate in human ccRCC tumors.

Analysis of the correlations between PBRM1 loss and p21 loss by Fisher's Exact tests in all samples (**top**), all grades (**middle**) and all stages (**bottom**).

		Fold Change (comparing to H1299-V)					
		H1299-p53		H1299-PBRM1-KO#1-V		H1299-PBRM1-KO#1-p53	
**Position**	**Symbol**	**Fold Change**	**Comments**	**Fold Change**	**Comments**	**Fold Change**	**Comments**
A01	APAF1	7.6683	OKAY	1.5862	OKAY	4.7453	OKAY
A02	ATM	1.3745	OKAY	1.2974	OKAY	1.2197	OKAY
A03	ATR	0.9006	OKAY	1.1216	OKAY	0.8625	OKAY
A04	BAI1	4.2249	A	1.1531	B	2.2138	B
A05	BAX	3.6023	OKAY	1.4903	OKAY	3.7751	OKAY
A06	BBC3	17.7396	A	11.8398	A	12.6102	A
A07	BCL2	0.8699	B	0.6716	B	0.6141	B
A08	BCL2A1	9.7737	B	1.1938	B	4.1598	B
A09	BID	2.7874	OKAY	1.7846	OKAY	2.3892	OKAY
A10	BIRC5	0.4115	OKAY	1.2532	OKAY	0.6627	OKAY
A11	BRCA1	0.4927	OKAY	1.2884	OKAY	0.6814	OKAY
A12	BRCA2	0.5137	OKAY	1.3524	OKAY	0.5891	OKAY
B01	BTG2	124.4053	A	1.8475	B	54.4369	A
B02	CASP2	0.5355	OKAY	0.7768	OKAY	0.5534	OKAY
B03	CASP9	1.1801	OKAY	1.6421	OKAY	1.4505	OKAY
B04	CCNB1	0.5066	OKAY	1.1531	OKAY	0.7054	OKAY
B05	CCNE1	1.3004	OKAY	0.5232	OKAY	1.0766	OKAY
B06	CCNG1	1.9574	OKAY	1.1294	OKAY	1.713	OKAY
B07	CCNH	0.8579	OKAY	1.0538	OKAY	0.9308	OKAY
B08	CDC25A	0.5942	OKAY	0.7986	OKAY	0.4312	OKAY
B09	CDC25C	0.3839	OKAY	1.6083	OKAY	1.0618	OKAY
B10	CDK1	0.4201	OKAY	1.025	OKAY	0.6582	OKAY
B11	CDK4	0.7214	OKAY	0.88	OKAY	0.7827	OKAY
B12	CDKN1A	97.6064	OKAY	1.0909	OKAY	45.7758	OKAY
C01	CDKN2A	0.6968	OKAY	1.2021	OKAY	0.9308	OKAY
C02	CHEK1	0.543	OKAY	1.4395	OKAY	0.8745	OKAY
C03	CHEK2	0.8943	OKAY	1.0321	OKAY	0.7666	OKAY
C04	CRADD	1.0345	B	0.85	B	0.5612	B
C05	DNMT1	0.5621	OKAY	0.9174	OKAY	0.6767	OKAY
C06	E2F1	0.5582	OKAY	1.1531	OKAY	0.7354	OKAY
C07	E2F3	0.9195	OKAY	0.4309	OKAY	0.4253	OKAY
C08	EGFR	1.1966	OKAY	1.8603	OKAY	2.1532	OKAY
C09	EGR1	0.9652	OKAY	0.7001	OKAY	0.7666	OKAY
C10	EI24	1.9847	OKAY	1.2274	OKAY	2.0944	OKAY
C11	ESR1	1.7519	B	1.0538	B	1.0328	B

C12	FADD	0.882	OKAY	0.9047	OKAY	0.6582	OKAY
D01	FAS	3.7293	OKAY	1.2021	OKAY	2.5964	OKAY
D02	FASLG	0.9992	C	1.3064	C	1.6206	C
D03	FOXO3	0.8943	OKAY	1.3713	OKAY	1.17	OKAY
D04	GADD45A	2.4604	OKAY	1.0685	OKAY	1.4404	OKAY
D05	GML	0.9992	C	1.3064	C	1.6206	C
D06	HDAC1	1.2217	OKAY	1.0611	OKAY	1.1539	OKAY
D07	HK2	0.806	OKAY	0.4749	OKAY	0.3833	OKAY
D08	IGF1R	2.8068	OKAY	1.8095	OKAY	3.1745	OKAY
D09	IL6	0.9992	C	1.3064	C	1.6206	C
D10	JUN	0.7469	OKAY	0.538	OKAY	0.3754	OKAY
D11	KAT2B	1.8776	OKAY	1.0538	OKAY	1.8875	OKAY
D12	KRAS	0.7949	OKAY	0.7714	OKAY	0.5973	OKAY
E01	MCL1	1.1164	OKAY	0.8441	OKAY	0.8745	OKAY
E02	MDM2	23.0853	OKAY	1.2359	OKAY	19.2464	OKAY
E03	MDM4	1.1087	OKAY	1.5216	OKAY	1.1619	OKAY
E04	MLH1	1.2388	OKAY	0.8985	OKAY	0.8331	OKAY
E05	MSH2	0.6502	OKAY	0.7608	OKAY	0.4495	OKAY
E06	MYC	0.7573	OKAY	0.8861	OKAY	0.7252	OKAY
E07	MYOD1	0.9992	C	1.3064	C	1.6206	C
E08	NF1	1.0273	OKAY	1.6883	OKAY	1.4404	OKAY
E09	NFKB1	1.3841	OKAY	0.8619	OKAY	0.9053	OKAY
E10	PCNA	1.2914	OKAY	0.6137	OKAY	0.9503	OKAY
E11	PIDD	10.7697	OKAY	0.6353	A	1.9139	OKAY
E12	PPM1D	2.3116	OKAY	1.1139	OKAY	1.7982	OKAY
F01	PRC1	0.5505	OKAY	1.0685	OKAY	0.5891	OKAY
F02	PRKCA	1.1801	OKAY	1.5216	OKAY	1.8233	OKAY
F03	PTEN	1.4131	OKAY	1.4495	OKAY	1.5872	OKAY
F04	PTTG1	1.0273	OKAY	1.2884	OKAY	1.2282	OKAY
F05	RB1	1.6574	OKAY	0.8985	OKAY	1.0917	OKAY
F06	RELA	0.8579	OKAY	1.0393	OKAY	1.0115	OKAY
F07	RPRM	8.5085	A	0.0441	B	1.2715	B
F08	SESN2	5.6919	OKAY	1.2619	OKAY	3.8278	OKAY
F09	SIAH1	0.7626	OKAY	1.1139	OKAY	0.9503	OKAY
F10	SIRT1	1.1011	OKAY	1.6766	OKAY	1.3072	OKAY
F11	STAT1	1.2474	OKAY	1.1692	OKAY	1.1459	OKAY
F12	TADA3	1.0345	OKAY	1.1856	OKAY	1.2029	OKAY
G01	TNF	0.9992	C	1.3064	C	1.6206	C
G02	TNFRSF10B	3.5038	OKAY	0.7248	OKAY	1.6777	OKAY
G03	TNFRSF10D	4.1093	OKAY	1.0909	OKAY	3.1745	OKAY
G04	TP53	73166.951	A	1.3064	C	276422.2	A
G05	TP53AIP1	0.9992	C	1.3064	C	1.6206	C
G06	TP53BP2	1.2388	OKAY	1.0465	OKAY	0.8745	OKAY
G07	TP63	0.852	B	1.1139	B	1.3818	B
G08	TP73	0.59	B	0.3312	B	0.2141	B

G09	TRAF2	0.6024	OKAY	1.0109	OKAY	0.6446	OKAY
G10	TSC1	1.0062	OKAY	1.4296	OKAY	1.1301	OKAY
G11	WT1	0.6638	OKAY	0.6137	OKAY	0.5093	OKAY
G12	XRCC5	0.9006	OKAY	1.0611	OKAY	0.8273	OKAY
H01	ACTB	0.9519	OKAY	0.963	OKAY	0.9771	OKAY
H02	B2M	1.1087	OKAY	1.6195	OKAY	1.6894	OKAY
H03	GAPDH	1.0859	OKAY	0.7148	OKAY	0.6861	OKAY
H04	HPRT1	0.882	OKAY	1.2884	OKAY	1.2453	OKAY
H05	RPLP0	0.9787	OKAY	0.8098	OKAY	0.8273	OKAY
H06	HGDC	0.9992	C	1.3064	C	1.6206	C
H07	RTC	1.3094	OKAY	1.2619	OKAY	1.3163	OKAY
H08	RTC	1.2474	OKAY	1.2189	OKAY	1.2627	OKAY
H09	RTC	1.3004	OKAY	1.2274	OKAY	1.1946	OKAY
H10	PPC	0.9787	OKAY	1.3524	OKAY	1.0692	OKAY
H11	PPC	0.9855	OKAY	1.3619	OKAY	1.1223	OKAY
H12	PPC	0.9519	OKAY	1.3431	OKAY	1.4505	OKAY

Supplementary Table 1. PCR array to examine the expression of most known p53 downstream targets in the parental and PBRM1 knockout (KO) H1299 cells after transfection of p53. Source data are provided as a Source Data file.
