# Supplementary material for: PBRM1 acts as a p53 lysine-acetylation reader to suppress renal tumor growth
Source: Nat Commun. 2019 Dec 20;10:5800. doi: 10.1038/s41467-019-13608-1 (PMC6925188; doi:10.1038/s41467-019-13608-1)
Supplement: Supplementary file 3 — Reporting Summary [file 41467_2019_13608_MOESM3_ESM.pdf]

nature research

Corresponding author(s): Haifeng Yang

Last updated by author(s): Aug 2, 2019

Reporting Summary

Nature Research wishes to improve the reproducibility of the work that we publish. This form provides structure for consistency and transparency in reporting. For further information on Nature Research policies, see Authors & Referees and the Editorial Policy Checklist .

Statistics

For all statistical analyses, confirm that the following items are present in the figure legend, table legend, main text, or Methods section.

n/a	Confirmed	
☐	☒	The exact sample size (*n*) for each experimental group/condition, given as a discrete number and unit of measurement
☐	☒	A statement on whether measurements were taken from distinct samples or whether the same sample was measured repeatedly
☐	☒	The statistical test(s) used AND whether they are one- or two-sided<br>*Only common tests should be described solely by name; describe more complex techniques in the Methods section.*
☐	☒	A description of all covariates tested
☐	☒	A description of any assumptions or corrections, such as tests of normality and adjustment for multiple comparisons
☐	☒	A full description of the statistical parameters including central tendency (e.g. means) or other basic estimates (e.g. regression coefficient) AND variation (e.g. standard deviation) or associated estimates of uncertainty (e.g. confidence intervals)
☐	☒	For null hypothesis testing, the test statistic (e.g. *F*, *t*, *r*) with confidence intervals, effect sizes, degrees of freedom and *P* value noted<br>*Give P values as exact values whenever suitable.*
☒	☐	For Bayesian analysis, information on the choice of priors and Markov chain Monte Carlo settings
☒	☐	For hierarchical and complex designs, identification of the appropriate level for tests and full reporting of outcomes
☒	☐	Estimates of effect sizes (e.g. Cohen's *d*, Pearson's *r*), indicating how they were calculated

Our web collection on statistics for biologists contains articles on many of the points above.

Software and code

Policy information about availability of computer code

Data collection	N/A
Data analysis	N/A

For manuscripts utilizing custom algorithms or software that are central to the research but not yet described in published literature, software must be made available to editors/reviewers. We strongly encourage code deposition in a community repository (e.g. GitHub). See the Nature Research guidelines for submitting code & software for further information.

Data

Policy information about availability of data

All manuscripts must include a data availability statement. This statement should provide the following information, where applicable:

- Accession codes, unique identifiers, or web links for publicly available datasets
- A list of figures that have associated raw data
- A description of any restrictions on data availability

Statistics source data for graphical representations and statistical analyses in Figs. 1-7, Supplementary Figs. 1-6 and Table S1 are provided in Source Data file. All the other data supporting the findings of this study are available from the corresponding author upon reasonable request.

Field-specific reporting

Please select the one below that is the best fit for your research. If you are not sure, read the appropriate sections before making your selection.

☒ Life sciences ☐ Behavioural & social sciences ☐ Ecological, evolutionary & environmental sciences

October 2018

Life sciences study design

All studies must disclose on these points even when the disclosure is negative.

Sample size	The size of xenograft analysis estimates are based on a two-sided t-test. Ten animals per group provides 80% power to detect differences in tumor volume of 0.133 cc between any two groups (5% level of significance), and is considered acceptable for these studies. No size analysis was performed for human ccRCC TMA analysis, but previous publications and statistical analysis revealed that samples from 160 patients are sufficient for statistical analysis.
Data exclusions	None.
Replication	Every exptertiment was repeated once or more to ensure every major finding is highly repeatable.
Randomization	Our samples are randomly picked as no filter was applied when deciding which animal or patient go into a group for comparison.
Blinding	When the human sample analysis was performed, neither the pathologist nor the statistician knew the identify of the samples or any expectation of the results.

Reporting for specific materials, systems and methods

We require information from authors about some types of materials, experimental systems and methods used in many studies. Here, indicate whether each material, system or method listed is relevant to your study. If you are not sure if a list item applies to your research, read the appropriate section before selecting a response.

Materials & experimental systems

n/a	Involved in the study	
☐	☒	Antibodies
☐	☒	Eukaryotic cell lines
☒	☐	Palaeontology
☐	☒	Animals and other organisms
☒	☐	Human research participants
☒	☐	Clinical data

Methods

n/a	Involved in the study	
☒	☐	ChIP-seq
☒	☐	Flow cytometry
☒	☐	MRI-based neuroimaging

Antibodies

Antibodies used	Antibodies for vinculin (sc-73614), actin (sc-8432), p53 (FL-393, sc-6243 and DO-1, sc-126) and p21 (sc-6246) were purchased from Santa Cruz Biotechnology. Antibodies for GST (#2625), p21 (#2947), PUMA (#12450) and Myc (#2040) were purchased from Cell Signaling Technology. Anti-PBRM1 (A301-591A), -BRD7 (A302-304A) and -BAF57 antibodies (A300-810A) were purchased from Bethyl Laboratories. Antibodies for Flag (M2, F3165), MDM2 (OP145) and γH2Ax (05-636) were purchased from Millipore Sigma. Anti-HA antibody (901514) was purchased from Biolegend. Anti-p53 K382Ac antibody (GTX62061) was purchased from GeneTex.
Validation	Antibodies against p53, p21, PBRM1, p53 K382Ac were validated with shRNA constructs against the target. The GST antibody was validated with lysates with without GST-fusion protein induced. The rest of proteins were validated by their respective cmpanies.

Eukaryotic cell lines

Policy information about cell lines

Cell line source(s)	HEK293, Caki-1 and ACHN cells were obtained from ATCC. HEK293T, RCC4, HCT-116, and NCI-H1299 were obtained from William Kaelin's lab at Dana-Farber Cancer Institute. Ren-01 cells were obtained from Danilel Lindner and Brian Rlni at Cleveland Clinic.
Authentication	HEK293, Caki-1 and ACHN cells were recently obtained from ATCC and it was authenticated there. The rest of the cell lines were not authenticated.
Mycoplasma contamination	All cell lines were examined with a kit of mycoplasma based on PCR detection. If any cell line is contaminated antibiotic treatment will be applied. All the cell lines were determined to be free from mycoplasma during experiments.
Commonly misidentified lines (See ICLAC register)	None of the cell line used belongs to the commonly misidentified lines.

Animals and other organisms

Policy information about studies involving animals; ARRIVE guidelines recommended for reporting animal research

Laboratory animals	4-6-week-old male Nu/J nude mice (Charles River).
Wild animals	None.
Field-collected samples	None.
Ethics oversight	All animal experiments were performed following the protocol 01462-935A approved by the Thomas Jefferson University Animal Care and Use Committee.

Note that full information on the approval of the study protocol must also be provided in the manuscript.
